# Pretreatment with β-Boswellic Acid Improves Blood Stasis Induced Endothelial Dysfunction: Role of eNOS Activation

**DOI:** 10.1038/srep15357

**Published:** 2015-10-20

**Authors:** Mingming Wang, Minchun Chen, Yi Ding, Zhihui Zhu, Yikai Zhang, Peifeng Wei, Jingwen Wang, Yi Qiao, Liang Li, Yuwen Li, Aidong Wen

**Affiliations:** 1 Department of pharmacy, Xijing Hospital, Fourth Military Medical University, Shaanxi, Xi’an 710032, China; 2 Shaanxi University of Chinese Medicine, Shaanxi, Xian-yang 712046, China

## Abstract

Vascular endothelial cells play an important role in modulating anti-thrombus and maintaining the natural function of vascular by secreting many active substances. β-boswellic acid (β-BA) is an active triterpenoid compound from the extract of boswellia serrate. In this study, it is demonstrated that β-BA ameliorates plasma coagulation parameters, protects endothelium from blood stasis induced injury and prevents blood stasis induced impairment of endothelium-dependent vasodilatation. Moreover, it is found that β-BA significantly increases nitric oxide (NO) and cyclic guanosine 3’, 5’-monophosphate (cGMP) levels in carotid aortas of blood stasis rats. To stimulate blood stasis-like conditions *in vitro*, human umbilical vein endothelial cells (HUVECs) were exposed to transient oxygen and glucose deprivation (OGD). Treatment of β-BA significantly increased intracellular NO level. Western blot and immunofluorescence as well as immunohistochemistry reveal that β-BA increases phosphorylation of enzyme nitric oxide synthase (eNOS) at Ser1177. In addition, β-BA mediated endothelium-dependent vasodilatation can be markedly blocked by eNOS inhibitor L-NAME in blood stasis rats. In OGD treated HUEVCs, the protective effect of β-BA is attenuated by knockdown of eNOS. In conclusion, the above findings provide convincing evidence for the protective effects of β-BA on blood stasis induced endothelial dysfunction by eNOS signaling pathway.

The gum resin of boswellia serrata, a kind of deciduous tree grown in dry parts of China and India, has been traditionally used for the treatment of inflammatory and arthritic diseases, prevention of myocardial ischemia reperfusion injury, and inhibition of platelet aggregation and extension of thrombin time^1^,^2^,^3^,^4^. β-boswellic acid (β-BA) is one of the most important active principles within the multicomponent mixture of boswellia serrata resin^5^,^6^. Recent studies have shown that post-treatment with 11-keto-β-boswellic acid or acetyl-11-keto-β-boswellic acid ameliorates cerebral ischemia–reperfusion injury^7^,^8^. However, little is known about the effects and molecular mechanisms on protecting endothelial function of both human and animal by β-BA.

Endothelium plays a predominant role in maintaining vascular homeostasis by means of modulation^9^. It has been demonstrated that blood stasis syndrome is closely related to hemorheological disorder, platelet dysfunction, vascular endothelium injury, microcirculation disturbance, inflammation and immunological regulation^10^. Endothelium is responsible for the maintenance of vascular homeostasis, thereby contributes to regulating vascular tone and thrombosis and also the proliferation and migration of smoothing muscle cell^11^,^12^. This balance may be disturbed and the endothelial cells are often damaged when disease arises, which could result in dysfunction and abnormal secretion of active substances (e.g. NO, NOS, ET-1, and prostacyclin PGI2)^13^.

Nitric oxide (NO), an antithrombotic product for endothelial cells, plays a crucial role in vascular homeostasis. It inhibits the aggregation of platelets and prevents adhesion of platelets to endothelium, and thereby prolongs the bleeding time^14^ and reduces the plasma levels of fibrinogen^14^. NO stimulates soluble guanylyl cyclase and increases concentrations of cyclic guanosine monophosphate (cGMP). NO and cGMP jointly comprise a special wide-ranging signal transduction system when multi roles of cGMP in physiological regulation are explored, including smooth muscle relaxation, visual transduction, intestinal ion transportation, and platelet function^15^,^16^. The constitutive calcium-calmodulin-dependent enzyme nitric oxide synthase (eNOS) is key for synthesization of NO from the amino acid L-arginine in endothelial cells^17^,^18^. It seems that phosphorylation of eNOS at Ser1177 is the most important site for eNOS activity regulation. Prevention of eNOS (Ser1177) phosphorylation by mutating the site to alanine reduces both basal and stimulated NO synthesization^19^. The anti-thrombotic effect of hydrogen sulfide is dramatically mediated by up-regulation of NO synthases. Inhibition of eNOS by L-NAME significantly reverts the antithrombotic effect of Na_2_S^20^. Related studies have shown that Fo Shou San (an ancient herbal decoction) can benefit endothelial function by increasing activity of eNOS^21^. As is suggested by recent studies, Gomisin J, a lignan from Schisandra chinensis, induces vascular relaxation via activation of eNOS in rat thoracic aorta endothelium^22^.

In order to explore the beneficial properties of boswellia serrata and the possible roles of eNOS-NO–cGMP pathway, it is hypothesized that β-BA can provide endothelium protection against vascular injury induced by blood stasis syndrome in rats and oxygen and glucose deprivation (OGD) in human umbilical vein endothelial cells (HUVECs).

## Results

### Effects of β-BA on plasma coagulation parameters

As is shown in Table 1, thrombin time (TT), prothrombin (PT) and activated partial thromboplastin time (APTT) were shortened, and fibrinogen (FIB) level was significantly increased in the model group. β-BA significantly prolonged TT, PT and APTT, and decreased FIB level compared with the model group, which demonstrated β-BA’s role to modulate plasma coagulation parameters in a dose-dependent manner.

### Effects of β-BA on *in vitro* endothelial function

**Figure 1:**
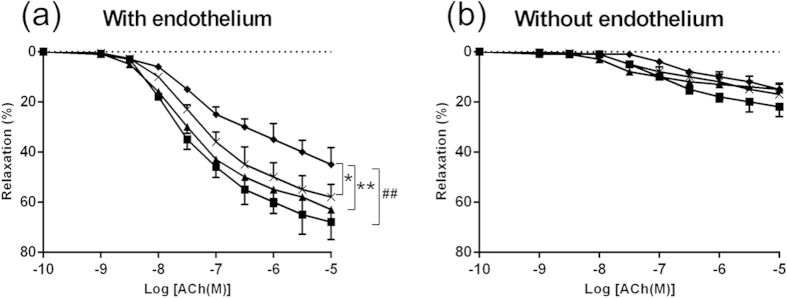
Effects of β-BA on ACh-mediated relaxation of mesenteric artery rings with or without endothelium. Mesenteric artery rings of blood stasis rats were isolated after oral administration with β-BA (100 mg/kg/d or 200 mg/kg/d) for 7 times. β-BA induced relaxation in aortic rings with (**a**) or without endothelium (**b**) was determined. The tension responsible for the vascular relaxation and constriction was tested. The contraction was induced by 0.5 mM phenylephrine. The results are expressed as percentage of relaxation for comparison with the remaining controlled tension (Mean ± SEM, n = 8). Experimental groups: control (■), model (⧫), model + β-BA 200 mg/kg (▲), model + β-BA 100 mg/kg (×). ^##^p < 0.01 versus the control group, ^#^p < 0.05 versus the control group, **p < 0.01 versus the model group, *p < 0.05 versus the model group.

Mesenteric artery rings from model animals showed weakened endothelium-dependent vasodilator response to acetylcholine in arteries stimulated by phenylephrine compared with control mesenteric artery rings^23^. Mesenteric artery rings from blood stasis treated animals showed reduced endothelium-dependent vasodilator responses to acetylcholine in artery rings stimulated by phenylephrine compared to control aortic rings ACh-mediated vessel relaxation was significantly improved in β-BA (100 mg/kg/d) and β-BA (200 mg/kg/d) groups compared with blood stasis rats (Fig. 1a). β-BA prevented the blood stasis induced impairment of endothelium-dependent vasodilatation. No differences were found among all experimental groups in the aspect of concentration–contractile response induced by phenylephrine in aortic rings without endothelium (Fig. 1b). However, the vasoconstrictor response to phenylephrine in intact mesenteric artery rings was increased by β-BA (Fig. 1a).

### Effects of β-BA on vascular endothelium of carotid aortas and HUVECs

**Figure 2:**
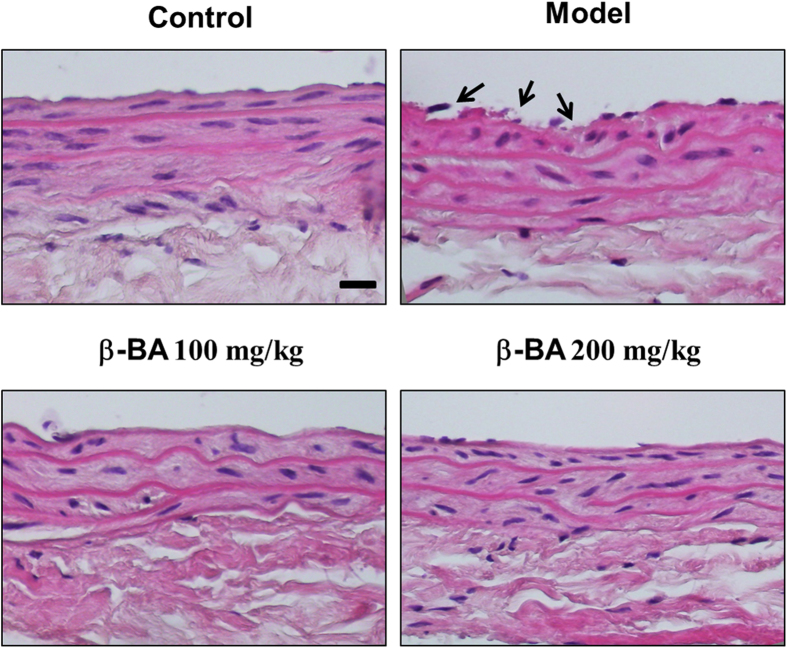
Representative images of HE staining performed on vascular endothelium in carotid aortas of blood stasis rats. The microscopic structures of carotid aortas were observed (bar: 100 μm). Arrows: broken endothelium in aorta.

**Figure 3:**
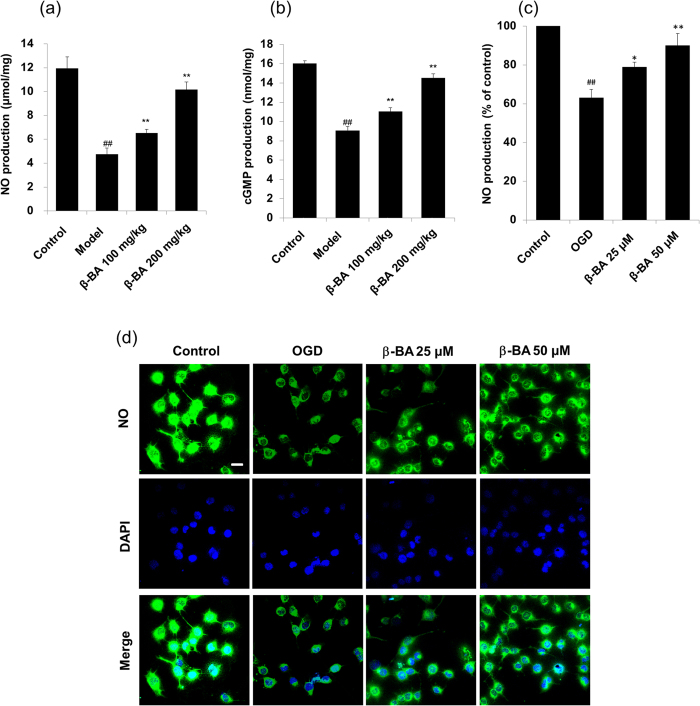
β-BA increased NO/cGMP levels in vascular endothelium of carotid aortas and HUVECs. After oral administration with β-BA (100 mg/kg/d or 200 mg/kg/d) for 7 times, NO (**a**) and cGMP (**b**) production in carotid arotas of rats were examined by Griess reaction and enzyme-linked immunosorbent assay. All data represent the results (Mean ± SD, n = 8). (^#^P < 0.05, ^##^P < 0.01 versus the control group. *P < 0.05, **P < 0.01 versus the model group). (**c**,**d**) HEUVCs were pretreated with β-BA for 24 h before being subjected to 6 h OGD then incubated with β-BA for an additional 24 h. NO production was measured by DAF-FM DA. The amount of NO was evaluated by measuring the fluorescence intensity excited at 495 nm and emitted at 515 nm. Representative images were taken by the confocal microscope (bar: 20 μm). All data represent the results (Mean ± SD) of triplicate independent experiments. ^##^p < 0.01 versus the control group, ^#^p < 0.05 versus the control group, **p < 0.01 versus OGD group, *p < 0.05 versus OGD group).

The H&E staining revealed that blood vessel endothelium in the normal group was integrated, but was not integrated in the model group. β-BA protected endothelium from the injury (Fig. 2). In addition, counts of circulating endothelial cells was performed. The results clearly showed that β-BA treatment significantly diminished circulating endothelial cells (CEC) count in blood compare to model (see Supplementary Fig. S1 online). The levels of NO and cGMP were determined in rats’ carotid aortas. β-BA significantly increased both the product of NO and cGMP in a dose-dependent manner (Fig. 3a,b). Moreover, NO production was directly investigated in cultured HUVECs by referring to NO indicator DAF-FM DA (Fig. 3c,d). Application of β-BA triggered a progressive rise in intracellular NO production in cultured HUVECs, as reflected by the increase of fluorescence intensity. The present results strongly indicate that β-BA could dose-dependently elevate the NO production in HUVECs.

### β-BA enhanced the phosphorylation of p-eNOS (Ser1177) in carotid aortas and HUVECs

**Figure 4:**
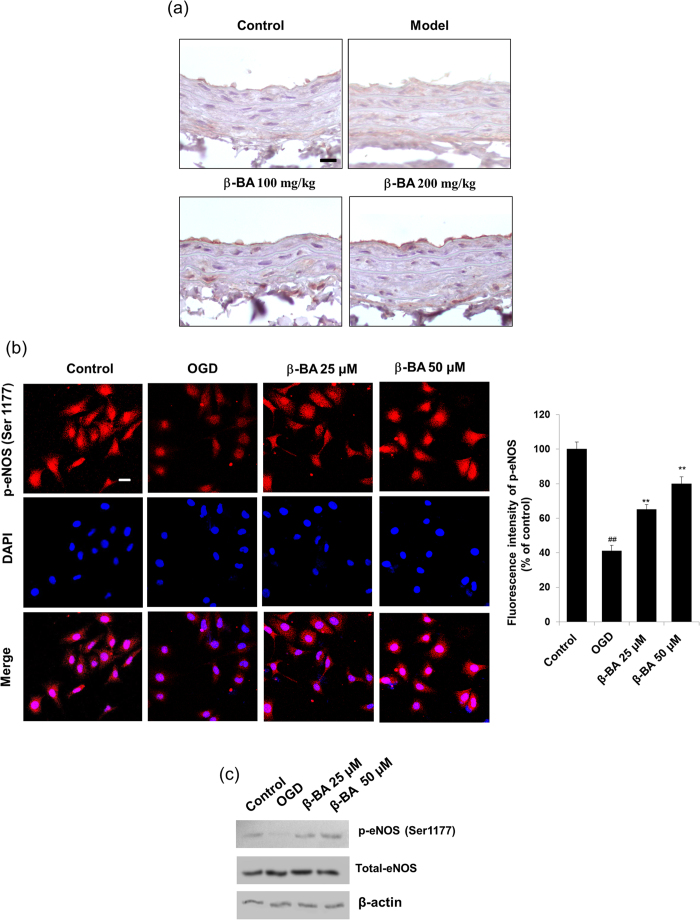
Effects of β-BA on the phosphorylation of eNOS at Ser1177 in carotid aortas and HUVECs. (**a**) After oral administration with β-BA (100 mg/kg/d or 200 mg/kg/d) for 7 times. Representative immunohistochemistry staining for p-eNOS (Ser1177) was shown on carotid arotas (bar: 100 μm); (**b**) HEUVCs were pretreated with β-BA for 24 h before being subjected to 6 h OGD then incubated with β-BA for an additional 24 h. Cells were immunostained with anti-phospho eNOS (Ser1177) antibodies, representative immunofluorescent staining for p-eNOS (Ser1177) was shown (bar 20 μm). DAPI: nuclear staining and merged images were also exhibited. Comparison of fluorescence intensity changes in HUVECs are presented by the histogram. (**c**) Western blot analysis showed the expression of p-eNOS (Ser1177) in HUVECs, and the blots were operated under the same experimental conditions. The data represent the results (Mean ± SD) of triplicate independent experiments.

As a key regulator of NO production, eNOS was investigated in terms of activity. Immunohistochemical analysis showed staining intensity of p-eNOS (Ser1177) in endothelium of carotid aorta. A significant reduction of p-eNOS (Ser1177) expression was displayed at the outer vascular endothelial cells in the model group, while β-BA markedly increased such expression, comparatively (Fig. 4a). Firstly, it has been demonstrated that 6 h OGD is enough to cause endothelial cell barrier dysfunction in HUVEC cells (see Supplementary Fig. S2 online). Then, expression of p-eNOS was also examined in HUEVCs under OGD, and it could be significantly enhanced by β-BA, as was displayed by immunofluorescence and western blot experiments (Fig. 4b,c).

### Phosphorylation of eNOS is essential for β-BA mediated protection of endothelium function

**Figure 5:**
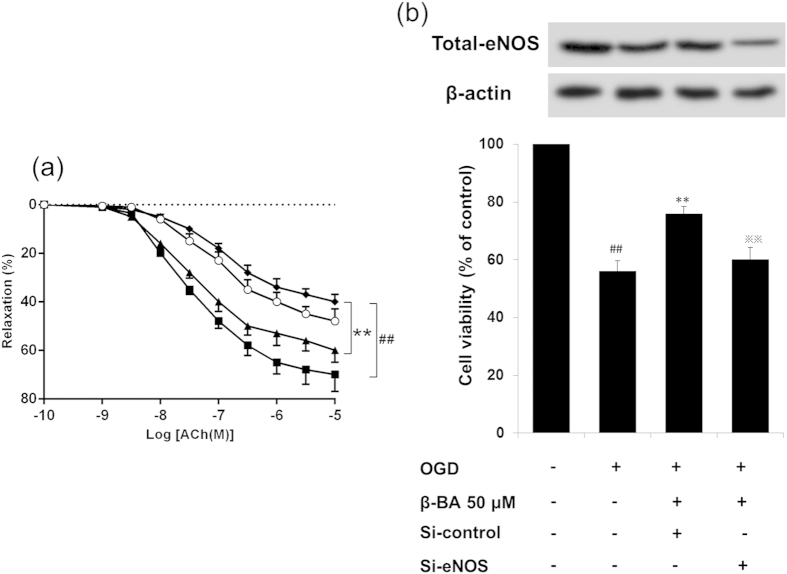
eNOS activity was essential for β-BA mediated relaxation of mesenteric artery rings and cell viability. (**a**) Representative tracings showed the vasorelaxant effects of β-BA (200 mg/kg/d) in phenylephrine pre-contracted aortic rings. β-BA induced relaxation could be significantly attenuated when the endothelium intact specimens were exposed to L-NAME (10^−4^ M). The values of results are expressed as Means ± SD (n = 8). Experimental groups: control (■), model (⧫), model + β-BA 200 mg/kg (▲), model + β-BA 200 mg/kg + L-NAME (○). ^##^p < 0.01 versus the control group, ^#^p < 0.05 versus the control group, **p < 0.01 versus the model group, *p < 0.05 versus the model group. (**b**) si-con and si-eNOS were transfected to HUEVCs for 12 h, then the cells were pretreated with β-BA for 24 h before being subjected to 6 h OGD then incubated with β-BA for an additional 24 h. Expression of eNOS was determined. Cell viability was measured with MTT assay. The data represent the results of (Mean ± SD) of triplicate independent experiments. ^##^p < 0.01 versus the control group, **p < 0.01 versus OGD group, 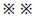
p < 0.01 versus β-BA group.

In the presence of L-NAME, the relaxation observed in response to the β-BA was significantly smaller than under control and β-BA (200 mg/kg) groups (Fig. 5a). Pretreatment of NO synthase inhibitor L-NAME reduced basal NO formation in the rats of the model group, treatment showed better contractile response in aorta compared with the model group, suggesting a higher NO formation in the vessel. eNOS phosphorylation and cell viability were increased by β-BA under OGD treatment in HUVECs (Fig. 5b), and the protective effect of β-BA was attenuated by knockdown of eNOS (Fig. 5b) (P < 0.01). All the aforementioned results indicate that eNOS is essential for β-BA mediated protection of endothelium function.

## Discussion

Boswellia serrata’s gum resin is one such plant used in Indian Ayurvedic and folk medicine to treat blood disorders and curtail inflammatory diseases like rheumatoid arthritis and to promote cardiac health^24^,^25^. Present study aims to investigate the mechanism of β-BA, an active triterpenoid compound from the extract of boswellia serrate, to protect endothelial function from blood stasis. Here, β-BA’s effective protection of endothelial function against blood stasis insult is firstly explained.

The blood stasis model was built during the time interval of two injections of adrenaline hydrochloride into the rats placed in ice-cold (0–2 °C) water. These data of blood coagulation parameters suggested that the injection of adrenaline hydrochloride and the exposure to ice-cold water could induce blood stasis^26^,^27^. The possibility to cause endothelial cell barrier dysfunction^28^ by OGD was demonstrated. HUVECs are suitable for studying endothelial barrier function because of their defined tight junction proteins^29^,^30^. Thus, *in vitro* endothelial barrier breakdown models were established in endothelial cell lines under OGD conditions. Since 6 h OGD destroys endothelial barrier function^28^ according to the results of relevant experiments, thus, 6 h OGD was selected to build endothelial barrier disruption models (Fig. 4).

As the key element in the interaction between blood flow and blood vessels, endothelial cell could modify a number of functions, such as vascular tension, platelet activity, tendency to thrombosis and fibrinolysis^31^. After being stained by H&E, the microscopic structures of rats’ carotid aortas were observed. Vascular endothelial cells of all administration treatment groups were protected (Fig. 2). Endothelial dysfunction characterized by a decrease in the bioavailability of vasodilator, like NO, as well as vascular complications have been observed in individuals^32^. In particular, as a cerebrovascular protector, endothelium-derived NO is considered as an important endogenous mediator of vascular homeostasis and blood flow^33^. The loss of endothelial NO impairs vascular function, partially by promoting vasoconstriction, platelet aggregation, smooth muscle cell proliferation, and leukocyte adhesion^34^. NO and cGMP jointly comprise a special wide-ranging signal transduction system when the multi roles of cGMP in physiological regulation are considered, including smooth muscle relaxation, visual transduction, intestinal ion transportation, and platelet function^35^. For example, increased cGMP in vascular smooth muscle cells underlying the endothelium activates GMP-dependent kinases that decrease intracellular calcium and producing relaxation^36^. β-BA treatment significantly increases NO and cGMP levels in both carotid aortas of blood stasis rats and OGD treated HUEVCs (Fig. 3).

Increased cGMP in platelets through action of NO released into the blood vessel lumen can decrease platelet activation and adhesion to the surface of endothelium^37^. NO can also regulate the cellular environment within the vessel wall by inhibiting the activity of growth factors released from cells within the vessel wall and from platelets on the endothelial surface^38^. Both water and hydroalcoholic extracts of boswellia serrata’s gum resin enhance PT and APTT coagulation time periods^39^. Extracts of boswellia serrata’s gum resin can be considered as an effective antiatherogenic resource for preventing coronary artery diseases and may serve as ideal source to isolate lead compounds of antiplatelet and anticoagulant therapeutics^39^. All the evidences raise the necessity to investigate the effects of β-BA on blood coagulation. As is manifested by the results, β-BA can significantly prolong TT, PT and APTT, and decrease FIB (Table 1). PT is referred to evaluate the overall efficiency of extrinsic clotting pathway, and prolonged PT indicates a deficiency in coagulation factors V, VII and X. On the other hand, APTT indicates the intrinsic clotting activity, and prolonged APTT usually represents a deficiency in factors VIII, IX, XI, XII and Von Willebrand’s factor^40^. According to the results, β-BA improves blood coagulation through extrinsic and intrinsic pathways. Additionally, it has been reported that β-BA induces release of arachidonic acids from platelets^41^, which in turn can induces endothelin-1 (ET-1) expression in endothelial cells^42^, Which has been identified as a key player of endothelial dysfunction. Pretreatment of β-BA results in a significant decrease of blood ET-1 level compared to model group (see Supplementary Fig. S3 online), which provides a better insight of β-BA’s protective mechanism.

In endothelial cells, NO is synthesized from substrate L-arginine via eNOS, and the phosphorylation of specific serine residue (Ser-1177) in eNOS is significant for its enzymatic activity^43^. Endothelium eNOS is the predominant isoform of NO synthase in vasculature and catalyzes the generation of NO^44^. Hallmark of a dysfunctional endothelium is an impaired action of the enzyme eNOS^45^. Western blot and immunofluorescence as well as immunohistochemistry revealed that β-BA could increase phosphorylation of eNOS at Ser1177 (Fig. 4). Endothelial dysfunction was mainly demonstrated by reduction of NO bioavailability^46^. In an isolated aortic ring, acetylcholine (ACh) induced endothelium-dependent relaxations, and the relaxations were abolished by eNOS inhibitor L-NAME^47^. NO formation was markedly reduced by L-NAME in blood stasis rats. β-BA treatment showed better contractile response in aorta compared with the model group, suggesting a higher NO formation in the vessel (Fig. 5a). Pretreatment with β-BA before OGD damage could significantly increase cell viability (Fig. 5b). However, the protective effect was reduced by knockdown of eNOS, which suggests eNOS is required for β-BA mediated endothelial protection.

In blood stasis rats, in OGD treated HUEVC cells, the protective effect of β-BA was attenuated by knockdown of eNOS. In conclusion, the findings convincingly support the protective effects of β-BA on blood stasis induced endothelial dysfunction by eNOS signaling pathway.

In summary, present study elucidates the cellular and molecular mechanisms of β-BA in blood vessels and human endothelial cells. Specifically, it is firstly demonstrated that β-BA can attenuate endothelial cells injury in blood stasis model, and protect HUVECs against OGD-induced cell death by activating the eNOS/NO/cGMP pathway. Collectively, it is proved that the unexplored potential of β-BA for the treatment of blood stasis damage and pharmacological activation of NO/cGMP pathway can ensure endothelial protection.

## Methods

### Animals and blood stasis syndrome model

Male Sprague-Dawley (SD) rats, weighing 220–280 g were supplied by the animal research center at Fourth Military Medical University, Xi’an, China. The experiments were performed in adherence with the National Institutes of Health Guidelines for the Use of Laboratory Animals and were approved by the Fourth Military Medical University Committee on Animal Care. β-BA (purity >98%) were purchased from the Chinese National Institute for the Control of Pharmaceutical and Biological Products (Beijing, China).

The Blood Stasis Syndrome model was produced as described previously^48^. Briefly, rats were kept in plastic cages at 22 ± 2 °C with free access to pellet food and water and on a 12 h light/dark cycle. Rats were randomly divided into four groups (control group, model group, model +β-BA100 mg/kg and model +β-BA 200 mg/kg group) with eight animals in each. Rats were given blank solvent as the vehicle at the same volume for control group. Rats were given blank solvent at the same volume for model group. In the model +β-BA (100 mg/kg) group, rats were given 100 mg/kg β-BA. In the model +β-BA (200 mg/kg) group, rats were given 200 mg/kg β-BA. All treatments were performed by gavage and were administered seven times with an interval of 12 h. After the fifth administration, the model rats except those in control group with blood stasis were established by being placed in ice-cold water (0–2 °C) for 5 min, during the interval between two injections of adrenaline hydrochloride (0.8 mg/kg). Rats were fasted overnight and administration continued after performing the model. Blood samples and carotid artery were collected 30 min after the last administration on the following day.

### Cell culture

HUVECs (ATCC, Manassas, VA, USA.) were cultured in DMEM (Gibco BRL, Grand Island, NY, USA) with 5 mM glucose and 10% fetal bovine serum (FBS) (Sijiqing, Hangzhou, China). Incubator containing 5% CO_2_ at 37 °C. Cells were sub-cultured when reaching 90% confluence.

### Cell Viability Assay

Cell viability was determined by a MTT [3- (4, 5-dimethylthiazol-2-yl) -2, 5-diphenyl-tetrazolium bromide] (Jiancheng, Nanjing, China) assay. Cells were seeded at a density of 1 × 10^4^ cells/well in 96-well cell culture plates. After treatment, 20 μl of the MTT solution (5 mg/ml) was added to each well (0.5 mg/ml final concentration in medium), and then the plates were incubated for an additional 4 h at 37 °C. Afterward the medium was removed and the metabolized MTT was solubilized with 150 μl DMSO. The absorbance of the solubilized blue formazan crystals was read at 490 nm. The percent viability was defined as the relative absorbance of treated versus that of untreated control cells.

### Oxygen glucose deprivation (OGD)

OGD was achieved using methods published^49^. Briefly, 24 h after HUVECs were seeded in different culture plates and the culture medium was changed to the glucose-free DMEM containing either β-BA at different final concentrations in 0.2% (w/v) DMSO in the β-BA -treated groups or 0.2% DMSO in the model-treated groups for 24 h. Then cells were placed into an anaerobic chamber that was flushed with 5% CO_2_ and 95% N_2_ (v/v). The cell cultures within the anaerobic chamber were kept in a humidified incubator at 37 °C for various time intervals in different experiments. To terminate the OGD, the culture medium was changed to normal medium containing the same concentration of β-BA in DMSO or DMSO alone before returning to the normoxic incubating conditions. In the control groups, the cell cultures were subjected to the same experimental procedures with vehicle only and without exposure to the glucose-free DMEM or anoxia.

### Plasma anticoagulation assay

Thrombin time (TT), prothrombin time (PT), activated partial thromboplastin time (APTT) and fibrinogen content (FIB) were examined with commercial kits following the manufacturer’s instructions by a coagulometer (Jiancheng, Nanjing, China). TT was determined by incubating 50 μl plasma solution for 3 min at 37 °C, followed by addition of 100 μl thrombin agent. PT was determined by incubating 50 μl plasma solution for 3 min at 37 °C, followed by addition of 100 μl thromboplastin agent. APTT was determined by incubating 50 μl plasma with 50 μl APTT activating agent for 3 min at 37 °C, followed by addition of 50 μl CaCl_2_. FIB was determined by incubating 10 μl plasma with 90 μl imidazole buffer for 3 min at 37 °C, followed by addition of 50 μl FIB agent. The anticoagulation activity was assessed by assaying the prolongation of the plasma clotting time of TT, PT, APTT, and reduction of FIB content.

### Vascular functional studies

One-millimeter ring segments of the mesenteric artery were dissected and mounted in individual organ chambers filled with Krebs buffer (composition in mM: NaCl 118, KCl 4.75, NaHCO_3_ 25, MgSO_4_ 1.2, CaCl_2_ 2, KH2PO4 1.2, glucose 11). The Krebs solution was continuously gassed with a 95% O_2_ and 5% CO_2_ mixture and kept at 37 °C. Rings were stretched to 2 g of resting tension by means of two L-shaped stainless-steel wires, which were inserted into the lumen and attached to the chamber and to an isometric force-displacement transducer, as previously described^50^. Rings were equilibrated for 60 to 90 min, and during this period, tissues were restretched and washed every 30 min with warm Krebs solution. Endothelial-dependent relaxation were assessed in response to increasing doses of acetylcholine (ACh) endothelium-dependent, after precontracted by 10^−6^ M phenylephrine. To evaluate the formation of basal NO, the contraction induced by 10^−6^ M phenylephrine was obtained in rings incubated for 30 min with the NOS inhibitor N^G^-nitro-L-arginine methyl ester (L-NAME, 10^−4^ M).

### Measurement of cGMP in carotid arteries

At the end of the experimental period, the carotid arteries was immediately isolated from a rat, and cut into segments of about 20 mg/tissue. The homogenate was centrifuged at 10,000 × g for 5 min, and the supernatant was removed and extracted three times with 1.5 ml of water-saturated diethyl ether. cGMP content was measured by the equilibrated radioimmunoassay as described previously^51^. In brief, standards or samples were introduced in a final volume of 100 μl of 50 mM sodium acetate buffer (pH 4.8). Then, 100 μl diluted cGMP antiserum and iodinated cGMP were added in succession and incubated for 24 h at 4 °C. The bound form was separated from the free form by charcoal suspension. Results were expressed as nanomole cGMP generated per milligram of protein (nmol/mg of protein).

### Measurement of NO in carotid arteries

NO Assay Kit (Jiancheng, Nanjing, China) was used to measure newly synthesized NO from L -arginine by the action of eNOS in the presence of essential cofactors, according to the manufacturer’s instructions. The final products of the reaction were nitrates, measured by colorimetric method (540 nm), which represented indirectly eNOS activity. Nitrate concentrations were determined via the standard curve.

### Measurement of NO in HUVECs

HUVECs (5 × 10^5^ cells/well) in 6-well plates were incubated with or without various concentrations of β-BA. The stimulated NO production was confirmed by laser confocal fluorescent microscopy using a specific dye: 4-amino-5-methylamino-20, 70-difluoro-fluorescein diacetate (DAF-FM DA) (Beyotime, Haimen, China). Optical density was read in a micro plate reader at 540 nm. Each experiment was performed in triplicate. Micrographs were taken by the confocal microscope.

### Histological and morphometric evaluations

Rats’ carotid aortas were isolated, fixed in formalin (10%), processed for paraffin sectioning (3 mm thick) and stained with hematoxylin-eosin (H&E). The lumen of blood vessels, vascular walls and vascular endothelial cells of the carotid aortas were observed with microscope.

### Immunohistochemical staining of rat’ carotid aortas endothelium

At the end of the experiments, rats’ carotid aortas were sampled and fixed in 4% phosphate buffered formaldehyde for 2 to 3 days^20^. After paraffin embedding tissue blocks were cut in 4 μm slices and tissue sections collected on poly-L-lysine-coated glass slides were treated by microwave for antigen unmasking. anti-eNOS (phospho Ser1177) antibody (Abcam, cambridge, UK) were used as primary antibodies at dilutions of 1:100 (eNOS) incubated overnight at 4 °C, followed by incubation with the appropriate secondary horseradish-peroxidase labeled antibodies in accordance to the instructions of the LSAB + System HRP kit (DAKO, Hamburg, Germany) and development using DAB as chromogen. The sections were examined by light microscopy (Zeiss Axioscop 40, Jena, Germany).

### Immunofluorescence

Cells grown in six-well slide chambers, after two washes with PBS, cells were fixed in 100% ethanol for 30 min. After 30 min of blocking of nonspecific binding with PBS containing 3% BSA, cells were incubated for 2 h at room temperature with a 1:100 dilution of the anti-eNOS (phospho Ser1177) antibody. After accurate washings, cells were further incubated for 1 h at room temperature with goat anti-rabbit IgG conjugated to Dylight 549 (diluted 1:100). After washing with PBS, Nuclei were incubated for 2 min at room temperature with DAPI (5 μg/ml). At the end, cells were washing twice with PBS, and cells were mounted in aqueous mounting medium and covered with coverslips. Specimens were evaluated with a microscope, and the images were captured using a Spot charge coupled device camera system.

### Western blot analysis

For western blot, equal amounts of protein lysates were separated using 10% sodium dodecyl sulfate-polyacrylamide (SDS-PAGE) gel electrophoresis. The gels were blotted onto a nitrocellulose membrane and incubated with the primary antibodies of phosphorylated-eNOS (Ser1177) and total eNOS (Abcam, cambridge, UK). Binding of primary antibody was detected with a secondary anti-rabbit antibody and visualized by the enhanced chemiluminescence method. β-actin was used as a loading control.

### Measurement of plasma endothelin-1 (ET-1) levels

The plasma of ET-1 were examined by ELISA (Enzyme-Linked Immunosorbent Assay) kit (Abcam, cambridge, UK). Blood samples were collected into plastic tubes containing EDTA (ethylenediaminetetraacetic acid), which were centrifuged at 3000 × g for 15 min, and the supernatant was assayed for the protein concentrations of ET-1 in accordance with the manufacturer’s instructions. The concentrations (pg/ml) were determined based on a standard curve, prepared using a known set of serial dilutions of standard proteins.

### Statistical Analysis

The statistical analyses were performed using SPSS 16.0 (SPSS Inc., Chicago, IL, USA). The results were expressed as Mean ± standard deviation (SD), and differences between groups were compared with one-way ANOVA or t-tests as appropriate. P-value less than 0.05 presented statistical significance.

## Additional Information

**How to cite this article**: Wang, M. *et al.* Pretreatment with β-Boswellic Acid Improves Blood Stasis Induced Endothelial Dysfunction: Role of eNOS Activation. *Sci. Rep.* **5**, 15357; doi: 10.1038/srep15357 (2015).

## Supplementary Material

Supplementary Information

## Figures and Tables

**Table 1:** Effect of β-BA on plasma coagulation parameters.

Group	Plasma coagulation parameters			
	TT (S)	PT (S)	APTT (S)	FIB (g/L)
Control	46.26 ± 2.17	13.10 ± 0.33	24.86 ± 0.47	1.79 ± 0.08
Model	29.56 ± 5.44##	8.60 ± 0.29##	17.48 ± 0.83##	4.05 ± 0.40##
β-BA (100 mg/kg)	34.81 ± 2.66**	9.17 ± 0.35**	18.41 ± 0.84*	3.48 ± 0.39**
β-BA (200 mg/kg)	44.27 ± 2.99**	11.55 ± 0.55**	22.87 ± 0.16**	2.49 ± 0.88**

TT, thrombin time; PT, prothrombin; APTT, activated partial thromboplastin time; FIB, fibrinogen. Date represent Mean ± SD, n = 8. ^##^p < 0.01 versus control group ^#^p < 0.05 versus control group ^**^p < 0.01 versus model group, ^*^p < 0.05 versus model group.
