# Supplementary material for: Pretreatment with β-Boswellic Acid Improves Blood Stasis Induced Endothelial Dysfunction: Role of eNOS Activation
Source: Sci Rep. 2015 Oct 20;5:15357. doi: 10.1038/srep15357 (PMC4611516; doi:10.1038/srep15357)
Supplement: Supplementary Information [file srep15357-s1.pdf]

Pretreatment with β-Boswellic Acid Improves Blood Stasis Induced Endothelial Dysfunction: Role of eNOS Activation

Mingming Wang[1,2+], Minchun Chen[1+], Yi Ding[1+], Zhihui Zhu[1+], Yikai Zhang[1], Peifeng Wei[2], Jingwen Wang[1], Yi Qiao[1], Liang Li[1], Yuwen Li[1#], Aidong Wen[1#]

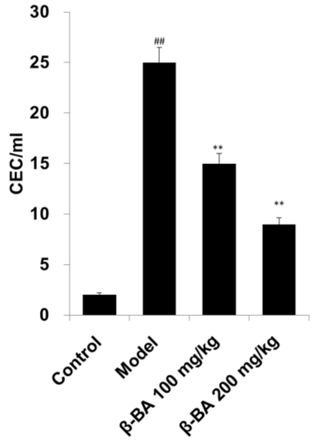

Figure S1. Counts of circulating endothelial cells (CEC) in rats. CEC counts per ml blood were shown. All data represent the Mean ± SD (n = 8). $^{\#}$, P < 0.05, $^{\#\#}$, P < 0.01 versus the control group; *, P < 0.05, **, P < 0.01 versus the model group.

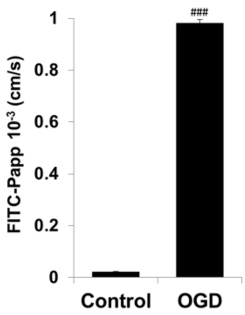

Figure S2. The effects of OGD induced increase of permeability in HUVECs. HUVECs were placed into an anaerobic chamber that was flushed with 5% CO_2 and 95% N_2 (v/v) condition for 6 h. Followed, epithelial barrier function was analyzed by Flux of FITC-conjugated dextran. Data represent the Mean ± SD from three independent experiments. $^{###}$, $p < 0.001$ versus control group. Papp: apparent permeability coefficient.

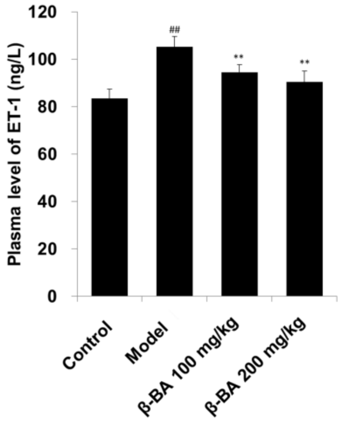

Figure S3. β-BA reduced the plasma ET-1 levels. After oral administration with β-BA (100 mg/kg/d or 200 mg/kg/d) for seven times, ET-1 productions in plasma of rats were examined by ELISA assay. All data represent the Mean ± SD (n = 8). $^{\#}$, P < 0.05, $^{\#\#}$, P < 0.01versus control group; *, P < 0.05, **, P< 0.01 versus model group.
